# Characterisation of *Ppy*-lineage cells clarifies the functional heterogeneity of pancreatic beta cells in mice

**DOI:** 10.1007/s00125-021-05560-x

**Published:** 2021-09-09

**Authors:** Takahiro Fukaishi, Yuko Nakagawa, Ayako Fukunaka, Takashi Sato, Akemi Hara, Keiko Nakao, Michiko Saito, Kenji Kohno, Takeshi Miyatsuka, Motoyuki Tamaki, Munehide Matsuhisa, Taka-aki Matsuoka, Tetsuya Yamada, Hirotaka Watada, Yoshio Fujitani

**Affiliations:** 1 Laboratory of Developmental Biology & Metabolism, Institute for Molecular and Cellular Regulation, Gunma University, Gunma, Japan; 2 Department of Molecular Endocrinology and Metabolism, Graduate School of Medical and Dental Sciences, Tokyo Medical and Dental University, Tokyo, Japan; 3 Department of Metabolism & Endocrinology, Juntendo University Graduate School of Medicine, Tokyo, Japan; 4 Center for Therapeutic Innovations in Diabetes, Juntendo University Graduate School of Medicine, Tokyo, Japan; 5 Department of Physiology, Faculty of Medicine, Saitama Medical University, Saitama, Japan; 6 Institute for Research Initiatives, Nara Institute of Science and Technology (NAIST), Nara, Japan; 7 Bio-science Research Center, Kyoto Pharmaceutical University, Kyoto, Japan; 8 Diabetes Therapeutics and Research Center, Institute of Advanced Medical Sciences, Tokushima University, Tokushima, Japan; 9 The First Department of Medicine, Wakayama Medical University, Wakayama, Japan; 10 Center for Identification of Diabetic Therapeutic Targets, Juntendo University Graduate School of Medicine, Tokyo, Japan; 11 Sportology Center, Juntendo University Graduate School of Medicine, Tokyo, Japan

**Keywords:** Beta cell heterogeneity, Ca^2+^ imaging, Diphtheria toxin, GLUT2, Pancreatic polypeptide, *Ppy*, Single-cell RNA sequence, Streptozotocin, TSPAN8, UCN3

## Abstract

**Aims/hypothesis:**

Pancreatic polypeptide (PP) cells, which secrete PP (encoded by the *Ppy* gene), are a minor population of pancreatic endocrine cells. Although it has been reported that the loss of beta cell identity might be associated with beta-to-PP cell-fate conversion, at present, little is known regarding the characteristics of *Ppy*-lineage cells.

**Methods:**

We used *Ppy-Cre* driver mice and a PP-specific monoclonal antibody to investigate the association between *Ppy*-lineage cells and beta cells. The molecular profiles of endocrine cells were investigated by single-cell transcriptome analysis and the glucose responsiveness of beta cells was assessed by Ca^2+^ imaging. Diabetic conditions were experimentally induced in mice by either streptozotocin or diphtheria toxin.

**Results:**

*Ppy*-lineage cells were found to contribute to the four major types of endocrine cells, including beta cells. *Ppy*-lineage beta cells are a minor subpopulation, accounting for 12–15% of total beta cells, and are mostly (81.2%) localised at the islet periphery. Unbiased single-cell analysis with a *Ppy*-lineage tracer demonstrated that beta cells are composed of seven clusters, which are categorised into two groups (i.e. *Ppy*-lineage and non-*Ppy*-lineage beta cells). These subpopulations of beta cells demonstrated distinct characteristics regarding their functionality and gene expression profiles. *Ppy*-lineage beta cells had a reduced glucose-stimulated Ca^2+^ signalling response and were increased in number in experimental diabetes models.

**Conclusions/interpretation:**

Our results indicate that an unexpected degree of beta cell heterogeneity is defined by *Ppy* gene activation, providing valuable insight into the homeostatic regulation of pancreatic islets and future therapeutic strategies against diabetes.

**Data availability:**

The single-cell RNA sequence (scRNA-seq) analysis datasets generated in this study have been deposited in the Gene Expression Omnibus (GEO) under the accession number GSE166164 (www.ncbi.nlm.nih.gov/geo/query/acc.cgi?acc=GSE166164).

**Graphical abstract:**

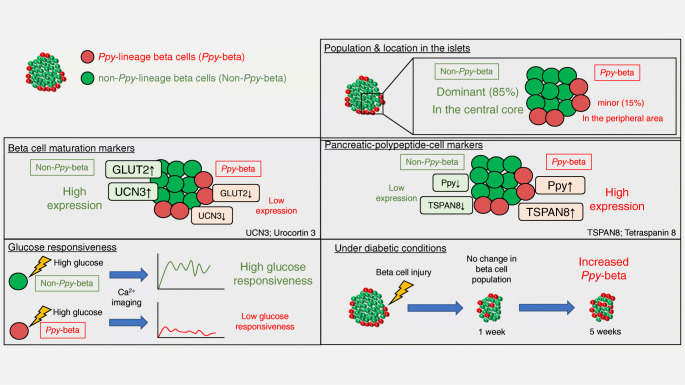

**Supplementary Information:**

The online version contains peer-reviewed but unedited supplementary material available at 10.1007/s00125-021-05560-x.

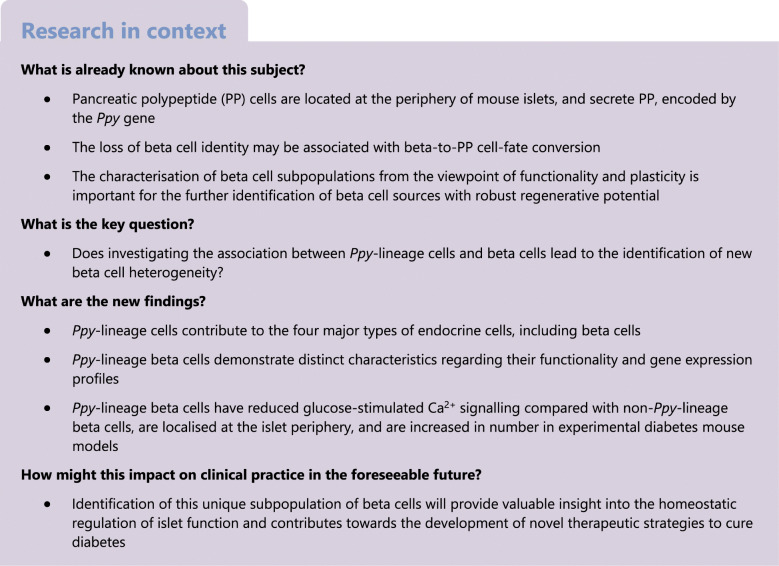



## Introduction

The islets of Langerhans consist of alpha, beta and delta cells and a fourth type of islet cell, namely, pancreatic polypeptide (PP) cells. PP cells are located at the periphery of the islets and secrete PP [1–3], encoded by the *Ppy* gene. PP is a member of the neuropeptide Y (NPY) family of peptides, which also includes peptide YY (PYY) and NPY, all reported to be involved in appetite regulation [4, 5]. However, the precise physiological functions of these peptides, including their roles in glucose homeostasis, remain poorly understood. A previous study demonstrated that *Ppy*-lineage cells were indispensable for the differentiation of a substantial fraction of endocrine cells by the diphtheria toxin (DT)-induced ablation of *Ppy*-lineage cells [6]. However, owing to the lack of specificity of the available PP antibody and the fidelity of the *Ppy* promoter used, the precise roles of *Ppy*-lineage cells have not yet been clarified.

Beta cell failure is the main hallmark of type 2 diabetes and it has been reported that inactivation of pancreatic and duodenal homeobox 1 (encoded by *Pdx1*) in mature beta cells results in the loss of beta cell identity and a beta-to-alpha cell fate conversion in vivo [7, 8]. Moreover, a reduction in the gene dose of *Pdx1* by heterozygous deletion of its evolutionarily conserved enhancer region caused an increase not only in the number of alpha cells but also in the number of PP cells in the pancreatic islets of adult mice, together with insufficient beta cell development [9]. In another report, *Rip-Cre-*mediated deletion of *Abcc8* in mice caused a sustained increase in intracellular Ca^2+^ concentrations, whereas beta cells underwent a fate switch to PP cells [10]. These findings suggest that unhealthy beta cells may shift not only towards alpha cells but also towards a PP cell fate.

Previous reports have highlighted the functional heterogeneity of beta cells. Many of these reports have particularly focused on the immature population of beta cells. For instance, they characterised immature beta cells located at the islet periphery, which are called ‘virgin beta cells’ [11], and low GLUT2-expressing immature beta cells with robust proliferative potential and resistance to streptozotocin (STZ)-induced cytotoxicity [12–16]. Other reports described Wnt-signal-regulated *Fltp* (also known as *Cfap126*)-positive beta cells [17] or NPY-positive immature beta cells [18]. The characterisation of beta cell subpopulations from the viewpoint of functionality and plasticity is especially important in the field of diabetes treatment for the further identification of beta cell sources with robust regenerative potential.

## Methods

### Animals

The study protocol was reviewed and approved by the Committee for Institutional Animal Care and Experimentation at Gunma University. All animals were housed in specific pathogen-free barrier facilities, maintained under a 12 h light/dark cycle, fed standard rodent food (CLEA Japan, Tokyo, Japan) and had access to water ad libitum. Male C57BL/6J mice (CLEA Japan, Tokyo, Japan) were used in all experiments, except for the single-cell RNA sequence (scRNA-seq) analysis and Ca^2+^ imaging experiments, in which male B6.Cg-Tg(*Ins1-EGFP*)1Hara/J (*MIP-GFP*) mice (stock number 006864; The Jackson Laboratory, Bar Harbor, ME, USA; https://www.jax.org/strain/006864) [19], which have a mixed background of CD-1 and C57BL/6J, were used. *Ppy-Cre* knockin mice were established previously (12-week-old male mice, weighing 23–26 g, were used), with the protein-coding region of *Ppy* in exon 2 being precisely replaced with that of NLS-Cre [20]. *Ppy-DTA* knockin mice, in which the diphtheria toxin A (DTA)-coding sequence was inserted in the *Ppy* locus, were also established by CRISPR/Cas9-mediated genome editing (12-week-old male mice, weighing 23–26 g, were used). *Ins-TR1* (C57BL/6-human *INS* promoter-DTR TRECK) transgenic mice [21] have been described previously. B6.129X1-*Gt(ROSA)26Sor*^*tm1(EYFP)Cos*^/J (*Rosa26-YFP*) mice (stock number 006148; The Jackson Laboratory; https://www.jax.org/strain/006148) and B6;129S6-*Gt(ROSA)26Sor*^*tm14(CAG-tdTomato)Hze*^/J (*Rosa26-tdTomato*) mice (stock number 007908; The Jackson Laboratory; https://www.jax.org/strain/007908) were used as reporter mice. Randomisation and blinding were not carried out in this study. Mice outside of the indicated weight range were excluded from the experiments. We repeated all experiments at least twice, except for scRNA-seq analysis, which was validated by additional immunohistochemistry. To perform the IPGTT, 2 g/kg of glucose was injected intraperitoneally into mice and blood glucose levels were measured at the indicated times.

### Immunohistochemistry and cell quantification

Harvested pancreases were divided into the head and tail and fixed in 4% vol./vol. paraformaldehyde at 4°C overnight. Then, pancreases were immersed in sucrose solution for 24 h prior to embedding in O.C.T compound (Sakura Finetek Japan, Osaka, Japan). Frozen pancreas blocks were sectioned at 14 μm thickness and immunostained. The following primary antibodies were used at the stated dilutions: guinea pig anti-insulin (INS; 1:100; DAKO, Glostrup, Denmark; catalogue no. IR002); mouse anti-glucagon (GCG; 1:1000; Abcam, Cambridge, UK; catalogue no. ab10988); rabbit anti-GCG (1:1000; Abcam; catalogue no. ab92517); rabbit anti-somatostatin (SST; 1:1000; Peninsula Laboratories, San Carlos, CA, USA; catalogue no. T-4103), mouse anti-PP (1:1000; IBL, Gunma, Japan; catalogue no. 23-2D3 [20]); chicken anti-green fluorescent protein (GFP; 1:1000; Abcam; catalogue no. ab13970); rabbit anti-chromogranin-A (1:100; Abcam; catalogue no. ab15160); rabbit anti-GLUT2 (1:200; Abcam; catalogue no. ab54460); rabbit anti-urocortin 3 (UCN3; 1:500; Phoenix Pharmaceuticals, Burlingame, CA, USA; catalogue no. H-019-29); rat anti-tetraspanin 8 (TSPAN8; 1:50; R&D Systems, Minneapolis, MN, USA; catalogue no. MAB6524); rabbit anti-folate receptor 1 (FOLR1; 1:100; Thermo Fisher Scientific, Waltham, MA, USA; catalogue no. PA5-42004); and rabbit anti-secreted phosphoprorein 1 (SPP1; 1:500; Sigma-Aldrich;, St Louis, MO, USA; catalogue no. AB10910). Primary antibodies were detected with the secondary antibodies listed in electronic supplementary material (ESM) Table 1, all used at 1:1000 dilution. DAPI (Dojindo, Kumamoto, Japan) was used for nuclear counterstaining. Fluorescence images were captured using an FV1000-D confocal microscope (Olympus, Tokyo, Japan). The total number of hormone- and yellow fluorescent protein (YFP)-positive cells in five to ten islets per pancreas from four mice per genotype were manually counted using Adobe Photoshop 2021 (Adobe, San Jose, CA, USA). Beta cell mass was measured as described previously [22].

### Diabetes induction

Diabetes was induced either by the injection of STZ (Sigma-Aldrich) into *Ppy-Cre;Rosa26-YFP* mice or by the injection of DT (Sigma-Aldrich) into *Ppy-Cre;Rosa26-YFP;Ins-TR1* mice. Blood glucose levels were monitored using Glutest mint (Sanwa Kagaku Kenkyusyo, Aichi, Japan). STZ was dissolved in citrate buffer (pH 4.5) prior to injection, and a single dose of 200 mg/kg was injected intraperitoneally into 6-week-old male mice (18–22 g). DT was dissolved in PBS prior to injection and a single dose of 100 ng/kg was injected intraperitoneally into 6-week-old male *Ppy-Cre;Rosa26-YFP;Ins-TR1* mice. Mice were killed at 7 days or 5 weeks after the injection.

### Ca^2+^ imaging

Islets were isolated from the pancreases of 8- to 10-week-old *MIP-GFP;Ppy-Cre;Rosa26-tdTomato* mice (22–25 g), as described previously [23]*.* Isolated beta cells were seeded onto poly-l-lysine-coated glass-bottomed dishes (MatTek Corporation, Ashland, MA, USA) 24 h before observation. Cells were washed with modified KRB buffer. Cells were loaded with 2 μmol/l Fura 2-AM (Dojindo, Kumamoto, Japan) and 0.01% vol./vol. Cremophor EL (Sigma-Aldrich) in a humidified incubator (95% air and 5% CO_2_ at 37°C) for 30 min. After washing with modified KRB, the cells were visualised using an Olympus UPlanAPO 10× water objective lens (Olympus). To obtain fluorescence images, the AQUACOSMOS/ASHURA 3CCD-based fluorescence energy transfer imaging system (Hamamatsu Photonics, Tokyo, Japan) was used. The ratio of 340/380 nm fluorescence was calculated and values were normalised to F0.

### Statistical analysis (other than for scRNA-seq analysis data)

Statistical analysis was performed using GraphPad Prism 9 software (GraphPad Software, San Diego, CA, USA). Data were expressed as means ± SEMs. Statistical comparisons between two groups were performed by the two-tailed Student’s *t* test, and one-way ANOVA followed by the Bonferroni post hoc test was used for comparisons between groups. A *p* value of less than 0.05 was considered to indicate a statistically significant difference between groups.

### scRNA-seq analysis

Islet cells were isolated from an 8-week-old male *MIP-GFP;Ppy-Cre;Rosa26-tdTomato* mouse. Cell quality was checked under a microscope before loading cells onto the chip, and cell viability was confirmed to be 84%. Cells that met one or more of the following three criteria, including doublets, were excluded from further analysis: (1) cells with 200 or less, or 8000 or more detected genes per cell; (2) cells with 80,000 or more unique molecular identifiers (UMIs) per cell; and (3) cells with 5% or more mitochondrial gene UMIs / the number of total gene UMIs per cell. Isolated single cells were loaded for scRNA-seq analysis using the Chromium System (10x Genomics, Pleasanton, CA, USA), following the manufacturer’s protocol of Single Cell 3′ Library kit v3.1. RNA libraries were sequenced on a Nova Seq6000 platform (Illumina, San Diego, CA, USA) with the following sequencing parameters: 28 bp read 1; 8 bp index 1; 91 bp read 2.

### Analysis of scRNA-seq data

Sequencing data were aligned to the mouse genome, Genecode release M25/GRCm38.p6 with GFP and tdTomato sequences, and UMI-collapsed with the Cell Ranger (v3.1.0) pipeline (10x Genomics).

For pre-processing of data, the Seurat (v3.1) R package for quality control filtering was used. Genes that were detected in at least three cells, and cells with more than 200 detected genes were selected. In addition, only cells with less than 8000 detected genes, less than 5% of mitochondrial genes, and less than 80,000 detected UMIs were retained.

Normalisation was performed using the global-scaling normalisation method, which normalises gene expression measurements for each cell by total gene expression, and then multiplies them by 10,000, and finally log-transforms the results. The variable genes were identified using the ‘vst’ method. Data were scaled to regress out the cell cycle score, the number of UMIs and the percentage of mitochondrial gene expression.

Based on the extracted variable genes, dimensionality reduction was performed by principal component analysis. A total of 50 dimensions were used for whole-cell analysis and 75 dimensions for beta cell analysis. Cell clustering was performed according to the Shared Nearest Neighbors method and was visualised in two-dimension by uniform manifold approximation and projection (UMAP).

Differentially expressed genes (DEGs) were identified by comparing the expression levels of the cluster cells with all other cells. For the analysis of statistical significance, the likelihood ratio test was performed by modelling as a two-part generalised regression model using model-based analysis of single-cell transcriptomics [24], and the Bonferroni method was used for multiple comparison adjustment. The mean fold change of expression was calculated by the following formula:

$$ \mathrm{Mean}\ \mathrm{fold}\ \mathrm{change}\ \mathrm{of}\ \mathrm{expression}=\left(\mathrm{mean}\ \mathrm{expression}\ \mathrm{of}\ \mathrm{the}\ \mathrm{cluster}+1\right)/\left(\mathrm{mean}\ \mathrm{expression}\ \mathrm{of}\ \mathrm{all}\ \mathrm{cells}\ \mathrm{other}\ \mathrm{than}\ \mathrm{the}\ \mathrm{cluster}+1\right) $$

## Results

### *Ppy*-lineage cells contribute to the four major types of endocrine cells

**Fig. 1:**
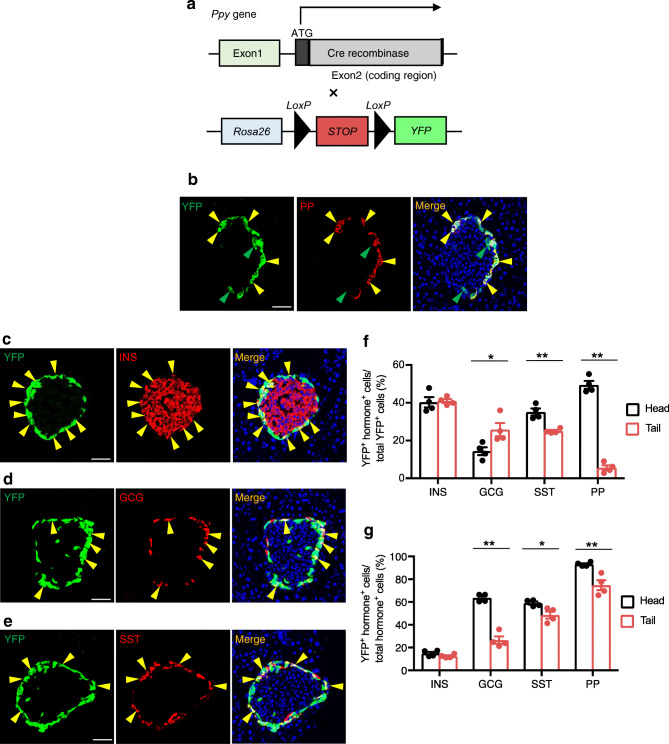
Analysis of *Ppy*-lineage cells using novel *Ppy*-*Cre* mice. (**a**) *Ppy-Cre* mice were crossed with *Rosa26-YFP* mice to generate *Ppy-Cre;Rosa26-YFP* mice. (**b**) YFP^+^ PP^+^ cells (yellow arrowheads) and YFP single-positive cells (green arrowheads) in the head of the pancreas of adult *Ppy-Cre;Rosa26-YFP* mice. Representative images of *n* = 4 mice. Scale bar, 50 μm. (**c**–**e**) YFP^+^ INS^+^ cells (**c**), YFP^+^ GCG^+^ cells (**d**) and YFP^+^ SST^+^ (**e**) double-positive cells in the head of the pancreas of adult *Ppy-Cre;Rosa26-YFP* mice (yellow arrowheads for all). Scale bars, 50 μm. (**f**) The ratio of YFP^+^ INS^+^/GCG^+^/SST^+^/PP^+^ (YFP^+^ hormone^+^) cells to total YFP^+^ cells in the pancreas of adult *Ppy-Cre;Rosa26-YFP* mice (*n* = 4). (**g**) The ratio of YFP^+^ hormone^+^ cells to each type of hormone^+^ cell in the pancreas of adult *Ppy-Cre;Rosa26-YFP* mice (*n* = 4). Data are shown as the mean ± SEM. *p < 0.05, **p < 0.01, (two-tailed Student’s *t* test)

The findings that the loss of beta cell identity may be associated with beta-to-PP cell-fate conversion led us to investigate the association between *Ppy*-lineage cells and beta cells. For this purpose, we analysed the fate of *Ppy*-expressing cells by developing a novel *Ppy*-knockin Cre driver, in which the coding region of the *Ppy* locus was replaced by that of Cre recombinase (Fig. 1a), and a PP-specific antibody (23-2D3) with no cross-reactivity to PYY or NPY [20]. In 12-week-old male *Ppy-Cre;Rosa26-YFP* mice, about 90% of the cells positive for PP encoded by the *Ppy* gene were positive for YFP, indicating high fidelity of this reporter (Fig. 1b). Notably, there were some YFP^+^ cells that were negative for PP (Fig. 1b, green arrowheads). Immunostaining analysis demonstrated that *Ppy*-lineage YFP^+^ cells contained cells that were costained for INS, GCG, SST or PP, indicating that *Ppy*-lineage cells contribute to all the four major types of endocrine cells (Fig. 1c–f). Some YFP^+^ GCG^+^ cells and YFP^+^ SST^+^ cells were also positive for PP, but there were very few YFP^+^ INS^+^ PP^+^ triple-positive cells (ESM Fig. 1a–c). The frequency of *Ppy*-lineage beta cells among total islet cells was similar between the head (14.9%) and tail (12.4%) of the pancreas, whereas the frequency of *Ppy*-lineage alpha (GCG^+^), delta (SST^+^) and PP cells differed between the two regions of the pancreas (Fig. 1g). We counted at least 1400 INS^+^ cells and 230 SST^+^ cells in the pancreatic head and tail of each mouse, and the number of each cell type was comparable between the two regions in each mouse. Importantly, we counted 365–489 GCG^+^ cells for each pancreatic tail but only 96–223 for each pancreatic head, and 258–436 PP^+^ cells for each pancreatic head but only 7–53 for each pancreatic tail. This difference in the population of endocrine cells between the head and tail is consistent with a previous report [25]. *Ppy*-lineage beta cells were mostly located at the peripheral region of the islets (81.2%), with a few cells in the central core of the islets (18.8%) (Fig. 1c and ESM Fig. 1a). As PP cells are much more abundant in the head of the pancreas, which is supposed to reflect the dynamics of *Ppy*-lineage and PP cells more accurately than the tail of the pancreas, we used this region for all subsequent analyses, unless otherwise stated.

### *Ppy-DTA* mice, which lack *Ppy*-lineage endocrine cells, demonstrate normal glucose tolerance

**Fig. 2:**
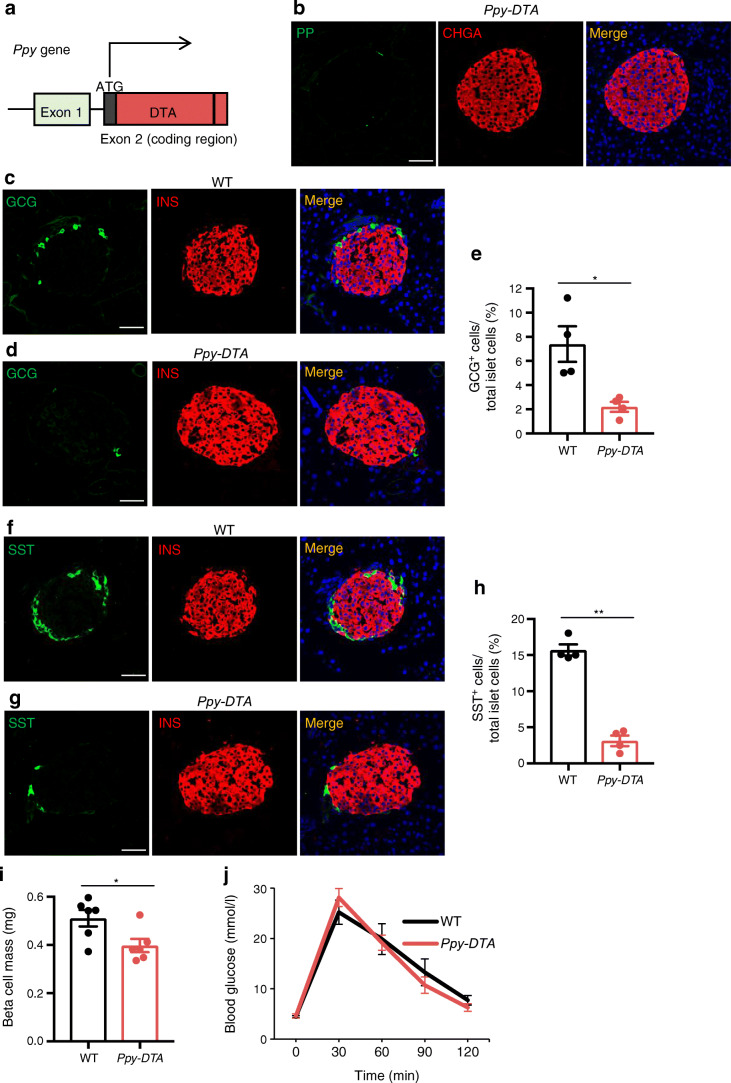
Analysis of *Ppy-DTA* mice. (**a**) Diagram of genotype of *Ppy-DTA* knockin mice. (**b**) Immunohistochemical analysis revealed almost complete deletion of PP cells in the head of the pancreas of adult *Ppy-DTA* mice. Representative images of *n* = 4 mice. CHGA, chromogranin-A. Scale bar, 50 μm. (**c**, **d**) Immunohistochemical analysis of INS^+^ and GCG^+^ cells in the head of the pancreas of adult WT mice (**c**) and *Ppy-DTA* mice (**d**). Representative images of *n* = 4 mice. Scale bars, 50 μm. (**e**) Ratio of GCG^+^ cells to total islet cells in adult WT vs *Ppy-DTA* mice (*n* = 4). (**f**, **g**) Immunohistochemical analysis of INS^+^ and SST^+^ cells in the head of the pancreas of adult WT mice (**f**) and *Ppy-DTA* mice (**g**). Representative images of *n* = 4 mice. Scale bars, 50 μm. (**h**) Ratio of SST^+^ cells to total islet cells in adult WT vs *Ppy-DTA* mice (*n* = 4). (**i**) Beta cell mass in the head of the pancreas of *Ppy-DTA* mice vs WT mice (*n* = 6). (**j**) Results of IPGTT performed on adult WT mice and *Ppy-DTA* mice. Data are shown as the mean ± SEM. *p < 0.05, **p < 0.01, (two-tailed Student’s *t* test)

We next investigated the physiological roles of *Ppy*-lineage cells and PP cells using 12-week-old male *Ppy-DTA* mice (Fig. 2a). In *Ppy-DTA* mice, *Ppy*-lineage cells are designed to express DTA, and these cells eventually die owing to the accumulation of DTA. The composition of islet cells in *Ppy-DTA* mice was significantly different from that of wild-type (WT) mice, particularly regarding non-beta endocrine cells. PP cells were almost completely absent in 12-week-old *Ppy-DTA* mice (Fig. 2b), and the ratio of alpha and delta cells to total islet cells was significantly lower in the head of the pancreas of *Ppy-DTA* mice than in WT mice (Fig. 2c–h). These data are consistent with the results that a substantial number of alpha and delta cells have a history of *Ppy* activation (Fig. 1g). Although beta cell mass was also significantly smaller in the head of the pancreas of *Ppy-DTA* mice compared with WT mice (Fig. 2i), *Ppy-DTA* mice showed normal glucose tolerance (Fig. 2j). This indicates that *Pp*y-lineage cells, including *Pp*y-lineage beta cells and PP cells, are dispensable for maintaining glucose tolerance at least in physiological conditions.

### scRNA-seq analysis clarified the heterogeneity of *Ppy* expression in beta cells

**Fig. 3:**
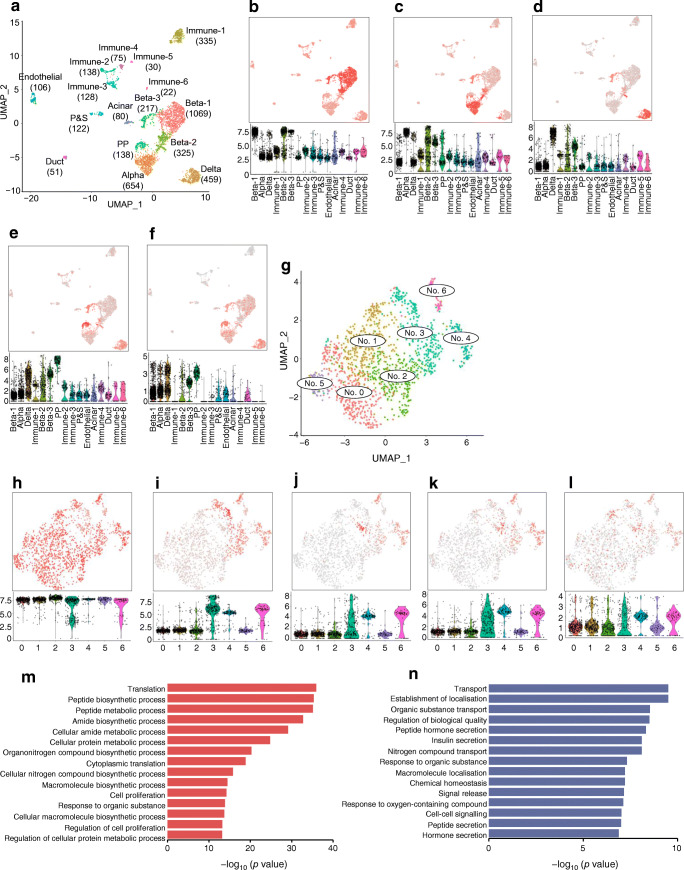
Single-cell transcriptome analysis of *Ppy*-lineage beta cells. (**a**) UMAP visualisation (in two dimensions [UMAP_1 and UMAP_2] of 3949 islet cells from an 8-week-old male *MIP-GFP;Ppy-Cre;Rosa26-tdTomato* mouse. Cell counts of each cluster are presented in brackets. (**b**–**f**) Feature plot and violin plot of *Ins2* (**b**), *Gcg* (**c**), *Sst* (**d**), *Ppy* (**e**) and *tdTomato* (**f**) mRNA expression in the various islet cell clusters. Relative expression index (arbitrary units) is shown on *y*-axes. (**g**) Reclustering of beta cells by UMAP visualisation identified seven subclusters (no. 0 to no. 6). (**h**–**l**) Feature plot and violin plot of *Ins2* (**h**), *Gcg* (**i**), *Sst* (**j**), *Ppy* (**k**) and *tdTomato* (**l**) mRNA expression in the various beta cell clusters shown in (**g**). Relative expression index (arbitrary units) is shown on *y*-axes. (**m**, **n**) GO analysis based on DEGs between *Ppy*-lineage and non-*Ppy*-lineage beta cells was performed using ShinyGO v0.61 Gene Ontology Enrichment Analysis. GO biological process output to comma-separated values files were saved, and all pathways with an adjusted *p* value of less than 0.01 were selected for the analysis. Commonly recurring and highly ranked GO pathways, which were highly enriched in genes derived from the differential gene expression analysis were selected. *p* values were normalised using the following formula: –log_10_(*p* value). GO pathways upregulated (**m**) and downregulated (**n**) in *Ppy*-lineage beta cells are shown. P&S, *Procr*^+^ and stellate

To investigate the molecular profiles of *Ppy*-lineage beta cells, we performed scRNA-seq analysis of islets (both the pancreatic head and tail) from an 8-week-old *MIP-GFP;Ppy-Cre;Rosa26-tdTomato* mouse. We profiled a total of 3949 cells using scRNA-seq analysis, and they were divided into 16 clusters (Fig. 3a–f). Each cluster was mapped to endocrine cells, exocrine cells or other known cell types. We also identified the recently described *Procr*^+^ progenitor cells, reproducing a recent study [26]. The PP cell population demonstrated the expression of unique signature genes, including the previously reported *Ppy*, *Pyy*, *Tspan8* and *Folr1* genes (ESM Fig. 2a, ESM Table 2) [26]. *tdTomato* was also detected as a marker gene for PP cells. Violin plots of *tdTomato* showed similar profiles to *Ppy* (Fig. 3e,f), corresponding to the *Ppy-Cre*-mediated activation of the *tdTomato* gene. Signals of *tdTomato* were detected not only in PP cells but also in alpha and delta cells (Fig. 3f). Therefore, the above data are consistent with the results that a significant fraction of alpha and delta cells have a history of *Ppy* activation (Fig. 1g).

To gain further insight into the heterogeneity of beta cells, transcriptomic characterisation of INS^+^ beta cells containing clusters beta-1, -2 and -3 was performed (see Fig. 3a). Refined clustering of beta cells demonstrated the presence of seven clusters (Fig. 3g–l, ESM Fig. 2b). In total, 24.6% of beta cells expressed *Ppy* mRNA and, importantly, such cells were enriched in clusters 4, 6 and 3, rather than being scattered among all beta cells, which is consistent with the enrichment of *tdTomato* mRNA (Fig. 3k,l). In cluster 3, however, the enrichment of *Ppy* and *tdTomato* mRNA was highly heterogeneous, in contrast to the homogeneous and clear enrichment of *Ppy* and *tdTomato* mRNA in clusters 4 and 6 (violin plots in Fig. 3k,l). For this reason, clusters 4 and 6 were annotated as *Ppy*-lineage beta cells and clusters 0, 1, 2 and 5 were annotated as non-*Ppy*-lineage beta cells in this study, and they were subjected to further analysis, including DEG and Gene Ontology (GO) analyses.

DEG analysis between *Ppy*-lineage and non-*Ppy*-lineage beta cells (ESM Table 3) demonstrated that *Ppy*-lineage beta cells show an upregulation of some of the signature genes for PP cells, including *Tspan8*, *Folr1*, *Spp1* and *Pyy* in addition to *Ppy*, indicating that these beta cells share molecular characteristics with canonical PP cells. Moreover, these beta cells showed the downregulation of key genes for beta cell maturation and INS secretion (e.g. *Slc2a2*, *Ucn3*, *Ins1*, *Ins2*, *MafA*, *Nkx6.1* [also known as *Nkx6-1*], *Neurod1*, *G6pc2, Sytl4* and *Ero1lb* [also known as *Ero1b*]; ESM Table 3). GO analysis of the DEGs identified the upregulation of several cell pathways, including the cell proliferation pathway, and the downregulation of the transport and INS secretion pathway in *Ppy*-lineage beta cells compared with non-*Ppy*-lineage beta cells (Fig. 3m,n).

### TSPAN8 is a marker for *Ppy*-lineage beta cells

**Fig. 4:**
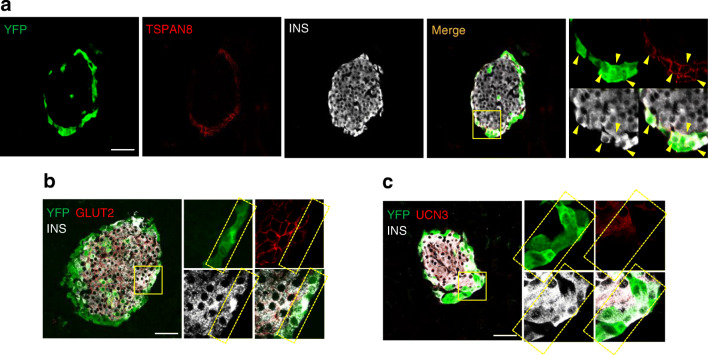
*Ppy*-lineage beta cells show higher expression of TSPAN8 and lower expression of GLUT2 and UCN3 than non-*Ppy*-lineage beta cells. (**a**) Immunohistochemical analysis of YFP^+^, TSPAN8^+^ and INS^+^ cells in the head of the pancreas of *Ppy-Cre;Rosa26-YFP* mice. Enlarged images of the inset are shown, with YFP^+^ TSPAN8^+^ INS^+^ cells indicated by arrowheads. Scale bar, 50 μm. (**b**) Immunohistochemical analysis of YFP^+^, GLUT2^+^ and INS^+^ cells in the head of the pancreas of *Ppy-Cre;Rosa26-YFP* mice. Enlarged images of the inset are shown, with YFP^+^ GLUT2^−^ INS^+^ cells indicated by the dotted area. Scale bar, 50 μm. (**c**) Immunohistochemical analysis of YFP^+^, UCN3^+^ and INS^+^ cells in the head of the pancreas of *Ppy-Cre;Rosa26-YFP* mice. Enlarged images of the inset are shown, with YFP^+^ UCN3^−^ INS^+^ cells indicated by the dotted area. Representative images of *n* = 4 mice. Scale bar, 50 μm

Next, to validate the molecular profiles of *Ppy*-lineage beta cells shown by scRNA-seq analysis at the protein level, we performed immunohistochemical analysis of some of the upregulated and downregulated markers extracted from DEG analysis and compared them in *Ppy*-lineage and non-*Ppy*-lineage beta cells. Among the markers analysed, we found that TSPAN8 was specifically expressed in *Ppy*-lineage beta cells. TSPAN8 is a member of the tetraspanin transmembrane protein family and has been reported as a PP-cell signature gene [26]. Approximately 40% of *Ppy*-lineage beta cells expressed TSPAN8, and, importantly, no expression was observed in non-*Ppy*-lineage beta cells (Fig. 4a).

As a negative marker, we found a low expression of GLUT2/*Slc2a2* in *Ppy*-lineage beta cells at the islet periphery (Fig. 4b). We also observed a lower expression of UCN3, a beta cell maturation marker, in *Ppy*-lineage beta cells compared with non-*Ppy*-lineage beta cells (Fig. 4c). These immunohistochemical results further confirm the PP-cell-like molecular characteristics and immaturity of *Ppy*-lineage beta cells indicated by scRNA-seq analysis.

### *Ppy*-lineage beta cells demonstrate reduced glucose-stimulated Ca^2+^ responses

**Fig. 5:**
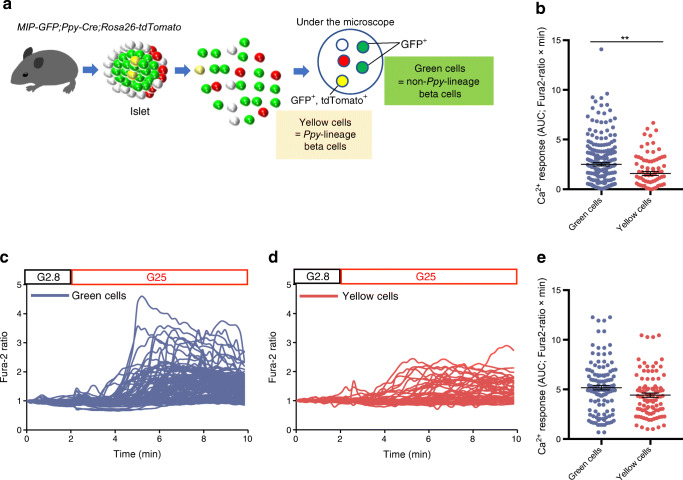
Comparison of glucose-stimulated Ca^2+^ influx between *Ppy*-lineage and non-*Ppy*-lineage beta cells. (**a**) Diagram of the method of identification of *Ppy*-lineage and non-*Ppy*-lineage beta cells using two reporters, GFP and tdTomato. (**b**) Ca^2+^ response to high-glucose stimulation in non-*Ppy*-lineage beta cells (green cells; *n* = 223) and *Ppy*-lineage beta cells (yellow cells; *n* = 84) from a total of *n* = 10 mice (*n* = 5 mice per group). Fura2-ratio is the ratio of 340/380 nm fluorescence. (**c**, **d**) Representative time course of Ca^2+^ responses in green cells (*n* = 111 cells) (**c**) and yellow cells (*n* = 44 cells) (**d**) from *n* = 1 mouse at glucose concentrations of 2.8 mmol/l (G2.8) and 25 mmol/l (G25). (**e**) Ca^2+^ response to 40 mmol/l K^+^ stimulation in non-*Ppy*-lineage beta cells (green cells; *n* = 62) and *Ppy*-lineage beta cells (yellow cells; *n* = 49) from a total of *n* = 8 mice (*n* = 4 mice per group). Data are shown as the mean ± SEM. **p < 0.01, (two-tailed Student’s *t* test)

Our scRNA-seq analyses demonstrated lower expression of a group of genes implicated in beta cell maturation and INS secretion in *Ppy*-lineage beta cells compared with non-*Ppy*-lineage beta cells (ESM Table 3). We hence postulated that *Ppy*-lineage beta cells may demonstrate decreased glucose responsiveness. To assess this, islets from 8- to 10-week-old *MIP-GFP;Ppy-Cre;Rosa26-tdTomato* mice were dispersed into single cells and then the increase in intracellular Ca^2+^ concentrations ([Ca^2+^]i), which is the final trigger of INS exocytosis, was measured in isolated non-*Ppy*-lineage (GFP^+^) and *Ppy*-lineage (GFP^+^ tdTomato^+^) beta cells during exposure to basal (2.8 mmol/l) and high (25 mmol/l) glucose concentrations (Fig. 5a). Single-cell Ca^2+^ imaging demonstrated that 25 mmol/l glucose increased [Ca^2+^]i in both beta cell types (Fig. 5b). The peak Ca^2+^ response was smaller in *Ppy*-lineage beta cells than in non-*Ppy*-lineage beta cells (Fig. 5c,d). Accordingly, the AUC of [Ca^2+^]i was significantly decreased in *Ppy*-lineage beta cells. A similar increase in [Ca^2+^]i was observed in both beta cell types when cells were depolarised by a 40 mmol/l K^+^ stimulation (Fig. 5e). Taken together, the results of our physiological studies demonstrated that *Ppy*-lineage beta cells show a smaller glucose-stimulated Ca^2+^ response, supporting our molecular findings.

### *Ppy*-lineage beta cells with low GLUT2 expression become dominant after STZ administration to mice

**Fig. 6:**
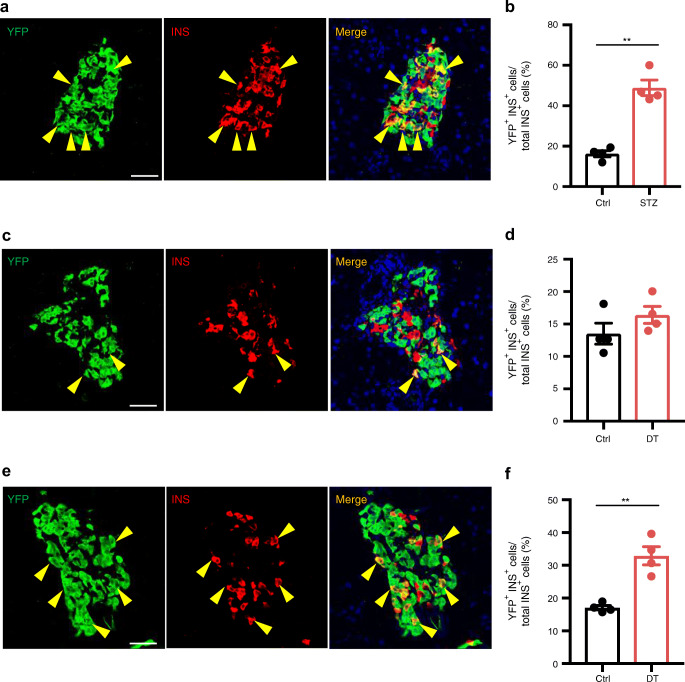
Characteristics of *Ppy*-lineage beta cells under hyperglycaemic conditions. (**a**) Immunohistochemical analysis of YFP^+^ INS^+^ cells (arrowheads) in the head of the pancreas of *Ppy-Cre;Rosa26-YFP* mice 7 days after 200 mg/kg STZ injection. Scale bar, 50 μm. (**b**) Ratio of YFP^+^ INS^+^ cells to total INS^+^ cells in the head of the pancreas of *Ppy-Cre;Rosa26-YFP* mice 7 days after 200 mg/kg STZ injection compared with control mice (*n* = 4). Control mice were treated with citrate buffer. (**c**) Immunohistochemical analysis of YFP^+^ INS^+^ cells (arrowheads) in the head of the pancreas of *Ppy-Cre;Rosa26-YFP;Ins-TR1* mice 7 days after 100 ng/kg DT injection. Scale bar, 50 μm. (**d**) Ratio of YFP^+^ INS^+^ cells to total INS^+^ cells in the head of the pancreas of *Ppy-Cre;Rosa26-YFP;Ins-TR1* mice 7 days after 100 ng/kg DT injection compared with control mice (*n* = 4). Control mice were treated with PBS. (**e**) Immunohistochemical analysis of YFP^+^ INS^+^ cells (arrowheads) in the head of the pancreas of *Ppy-Cre;Rosa26-YFP;Ins-TR1* mice 5 weeks after 100 ng/kg DT injection. Scale bar, 50 μm. (**f**) Ratio of YFP^+^ INS^+^ cells to total INS^+^ cells in the head of the pancreas of *Ppy-Cre;Rosa26-YFP;Ins-TR1* mice 5 weeks after 100 ng/kg DT injection compared with control mice (*n* = 4). Control mice were treated with PBS. Ctrl, control mice. Data are shown as the mean ± SEM. **p < 0.01 (two-tailed Student’s *t* test)

To characterise the behaviour of *Ppy*-lineage beta cells in diabetes, a single dose of 200 mg/kg STZ was administered to 6-week-old male *Ppy-Cre;Rosa26-YFP* mice. Mice were killed after 7 days and their pancreatic tissue was analysed. Mice became hyperglycaemic shortly after STZ injection and *Ppy*-lineage beta cells became relatively dominant (Fig. 6a,b), accounting for 48.8% of the remaining beta cells. TSPAN8^+^ INS^+^ beta cells also became dominant (ESM Fig. 3a–c), consistent with the results shown in Fig. 4a. However, the induction of hyperglycaemia by the administration of DT to 6-week-old male *Ppy-Cre;Rosa26-YFP;Ins-TR1* mice, in which beta cells can be ablated by the administration of DT acting on the DT receptors that they express, did not change the proportion of *Ppy*-lineage beta cells among the remaining beta cells 7 days after DT administration (Fig. 6c,d). Considering that STZ is transported into beta cells via GLUT2, the increased population of *Ppy*-lineage beta cells in the islets of mice with STZ-induced diabetes is thought to correspond to the *Ppy*-lineage beta cells with low expression of *Slc2a2*/GLUT2 observed by scRNA-seq analysis and immunohistochemistry. Intriguingly, *Ppy*-lineage beta cells became dominant also in *Ppy-Cre;Rosa26-YFP;Ins-TR1* mice 5 weeks after DT administration (Fig. 6e,f), suggesting that *Ppy*-lineage beta cells are more resistant to prolonged hyperglycaemia than non-*Ppy*-lineage beta cells.

## Discussion

In this study, lineage tracing and scRNA-seq analysis using lineage tracers were performed to characterise PP cells and *Ppy*-lineage cells. YFP and *tdTomato* were detected in alpha and delta cells, in addition to beta cells (Figs 1c–e, 3f), suggesting that a substantial fraction of endocrine cells have a history of *Ppy* gene activation. Indeed, scRNA-seq analysis of adult WT islets showed that a subpopulation of alpha, beta and delta cells express *Ppy* mRNA, and the coexpression of PP and other endocrine hormones in these cells was confirmed at the protein level (ESM Fig. 1a–c). The substantial reduction of alpha and delta cell numbers in *Ppy-DTA* mice (Fig. 2c–h) also supports the idea that activation of the *Ppy* gene occurs in these endocrine cell types.

*Ppy*-lineage cells can contribute to all four major types of endocrine cells. The results of cell-lineage tracing may simply reflect the heterogeneity of beta cells, in which a subpopulation of beta cells express *Ppy*. Beta cell heterogeneity has attracted much attention and provides additional insight into the homeostatic regulation of islet function in the progression and treatment of diabetes. ‘Virgin beta cells’ [11] showed similar characteristics to the *Ppy*-lineage beta cells, with low levels of GLUT2 expression and their localisation at the islet periphery. However, these beta cells were shown to be induced via alpha-to-beta transdifferentiation, as assessed by *Gcg-Cre*-mediated lineage tracing. Therefore, ‘virgin beta cells’ were considered as a neogenic niche, a continuous supply of cells provided from alpha cells. There are reports of other beta cell subpopulations showing low levels of GLUT2 expression, robust proliferative capacity and resistance to STZ-induced cytotoxicity [12–16], possibly sharing the characteristics of *Ppy*-lineage beta cells shown in the present study (i.e. immaturity and impaired function confirmed by the low expression of GLUT2 and UCN3 and a low glucose-stimulated Ca^2+^ response). However, these previous studies lacked analysis using a molecular marker that identifies specific types of beta cells, and we propose that *Ppy* gene expression is a candidate for such a marker.

Additional analysis of the pathophysiological characteristics of *Ppy*-lineage beta cells in diabetic conditions using *Ppy-CreERT2* knockin mice, which we generated together with *Ppy*-Cre knockin mice (data not shown), is expected to provide further useful information. However, as the pulse-and-chase labelling efficiency of these mice (particularly in their *Ppy*-lineage beta cells) is low, it is difficult to analyse the dynamics of *Ppy*-lineage beta cells within a specific time window in these mice. This insufficient labelling efficiency of *Ppy*-lineage beta cells might be owing to suppressed activity of the *Ppy* promoter during their differentiation into beta cells. The establishment of tools and techniques for detecting low levels of *Ppy* gene transcription in endocrine cells within a specific time window will resolve this limitation of our present study.

In this study, we found that 40% of the *Ppy*-lineage beta cells express TSPAN8 (Fig. 4a), a member of the tetraspanin transmembrane protein family that is mainly expressed in the gastrointestinal tract in both mice and humans [27]. *Tspan8* has been reported as a PP-cell signature gene [26] that is highly expressed in human pancreatic ductal progenitors [28]. Other PP-cell signature markers, such as PP (ESM Fig. 1a), FOLR1 and SPP1 (ESM Fig. 4a,b), were rarely merged with *Ppy*-lineage beta cells [26, 29]. Some recent reports showed that TSPAN8 regulates cell proliferation, invasion and metastasis in various types of tumours, including pancreatic adenocarcinoma [30]. Further investigation of the physiological role of TSPAN8 in *Ppy*-lineage beta cells will also clarify the physiological role of *Ppy*-lineage beta cells, particularly those associated with their proliferative characteristics. Regarding cell proliferation, DEG analysis identified the upregulation of cell proliferation markers in *Ppy*-lineage beta cells (e.g. *Jun*, *Junb*, *Fyn*, *Fgfr1*, *Pdgfb* and *Reg1*). Of these enriched genes, *Pdgfb* and *Reg1* are of particular interest, as they have been reported to regulate beta cell proliferation during ageing and in some models of diabetes [31–34]. The upregulation of these genes associated with beta cell proliferation indicates that *Ppy*-lineage beta cells are a promising therapeutic target in diabetes.

In summary, we found an unexpected degree of beta cell heterogeneity while investigating the characteristics of *Ppy*-lineage cells. High-resolution single-cell transcriptome analysis demonstrated that this subpopulation of beta cells shows unique functional characteristics and gene expression profile. We can speculate that *Ppy*-lineage beta cells with low levels of GLUT2 and UCN3 expression may be generated by the pancreas to survive conditions of metabolic stress under hyperglycaemia, at the expense of glucose-induced INS secretion. Identification of this unique subpopulation of beta cells is expected to provide valuable insight into the homeostatic regulation of islet function and contribute towards the development of novel therapeutic strategies to cure diabetes.

## Supplementary Information

ESM (PDF 1.68 mb)

ESM Table 2 (XLSX 3656 kb)

ESM Table 3 (XLSX 86 kb)

## Data Availability

The scRNA-seq analysis datasets generated in this study have been deposited in the Gene Expression Omnibus (GEO) under the accession number GSE166164 (www.ncbi.nlm.nih.gov/geo/query/acc.cgi?acc=GSE166164). Other datasets are available from the corresponding author upon reasonable request.

## Abbreviations

[Ca^2+^]i: Intracellular Ca^2+^ concentration
DEG: Differentially expressed gene
DT: Diphtheria toxin
DTA: Diphtheria toxin A
FOLR1: Folate receptor 1
GCG: Glucagon
GFP: Green fluorescent protein
GO: Gene ontology
INS: Insulin
NPY: Neuropeptide Y
PP: Pancreatic polypeptide
PYY: Peptide YY
scRNA-seq: Single-cell RNA sequence
SPP1: Secreted phosphoprotein 1
SST: Somatostatin
STZ: Streptozotocin
TSPAN8: Tetraspanin 8
UCN3: Urocortin 3
UMAP: Uniform manifold approximation and projection
UMI: Unique molecular identifiers
WT: Wild-type
YFP: Yellow fluorescent protein
