# Supplementary material for: Characterisation of Ppy-lineage cells clarifies the functional heterogeneity of pancreatic beta cells in mice
Source: Diabetologia. 2021 Sep 9;64(12):2803–16. doi: 10.1007/s00125-021-05560-x (PMC8563568; doi:10.1007/s00125-021-05560-x)
Supplement: Supplementary file 1 — (PDF 1.68 mb) [file 125_2021_5560_MOESM1_ESM.pdf]

ESM Table 1 The combinations of primary antibodies and secondary antibodies

Primary antibody	Secondary antibody
guinea-pig anti-insulin (DAKO, catalogue no. IR002)	Goat anti-guinea pig IgG (H+L) secondary antibody, Alexa Fluor 594 (Thermo Fisher Scientific, catalogue no. A11076)
	Goat anti-guinea pig IgG (H+L) secondary antibody, Alexa Fluor 647 (Thermo Fisher Scientific, catalogue no. A21450)
mouse anti-glucagon (Abcam, catalogue no. ab10988)	Goat anti-mouse IgG(H+L) secondary antibody, Alexa Fluor 488 (Thermo Fisher Scientific, catalogue no. A11001)
	Goat anti-mouse IgG(H+L) secondary antibody, Alexa Fluor 594 (Thermo Fisher Scientific, catalogue no. A11005)
rabbit anti-glucagon (Abcam, catalogue no. ab92517)	Goat anti-rabbit IgG H&L secondary antibody, Alexa Fluor 405 (Abcam, catalogue no. ab175652)
rabbit anti-somatostatin (Peninsula Laboratories, catalogue no. T-4103)	Goat anti-rabbit IgG (H+L) secondary antibody, Alexa Fluor 594 (Thermo Fisher Scientific, catalogue no. A11012)
	Donkey anti-rabbit IgG (H+L) secondary antibody, Alexa Fluor 488 (Thermo Fisher Scientific, catalogue no. A21206)
	Goat anti-rabbit IgG H&L secondary antibody, Alexa Fluor 405 (Abcam, catalogue no. ab175652)
mouse anti-PP (IBL, catalogue no. 23-2D3)	Goat anti-mouse IgG(H+L) secondary antibody, Alexa Fluor 488 (Thermo Fisher Scientific, catalogue no. A11001)
	Goat anti-mouse IgG(H+L) secondary antibody, Alexa Fluor 594 (Thermo Fisher Scientific, catalogue no. A11005)
chicken anti-GFP (1:1000) (Abcam, catalogue no. ab13970)	Goat anti-chicken IgG (H+L) secondary antibody, Alexa Fluor 488 (Thermo Fisher Scientific, catalogue no. A11039)
rabbit anti-chromogranin-A (Abcam, catalogue no. ab15160)	Goat anti-rabbit IgG (H+L) secondary antibody, Alexa Fluor 594 (Thermo Fisher Scientific, catalogue no. A11012)
rabbit anti-GLUT2 (Abcam, catalogue no. ab54460)	Goat anti-rabbit IgG (H+L) secondary antibody, Alexa Fluor 594 (Thermo Fisher Scientific, catalogue no. A11012)
rabbit anti-UCN3 (Phoenix Pharmaceuticals, catalogue no. H-019-29)	Goat anti-rabbit IgG (H+L) secondary antibody, Alexa Fluor 594 (Thermo Fisher Scientific, catalogue no. A11012)
rat anti-TSPAN8 (R&D Systems, catalogue no. MAB6524)	Goat anti-rat IgG(H+L) secondary antibody, Alexa Fluor 488 (Thermo Fisher Scientific, catalogue no. A11006)
	Goat anti-rat IgG H&L secondary antibody, Alexa Fluor 594 (Abcam, catalogue no. ab150160)
rabbit anti-FOLR1 (Thermo Fisher Scientific, catalogue no. PA5-42004)	Goat anti-rabbit IgG (H+L) secondary antibody, Alexa Fluor 594 (Thermo Fisher Scientific, catalogue no. A11012)
rabbit anti-SPP1 (Sigma-Aldrich, catalogue no. AB10910)	Goat anti-rabbit IgG (H+L) secondary antibody, Alexa Fluor 594 (Thermo Fisher Scientific, catalogue no. A11012)

ESM Table 2 (Differentially expressed genes in each of the 16 islet cell clusters) and Table 3 (Differentially expressed genes analysis between Ppy-lineage and non-Ppy-lineage beta cells) are provided as Excel files.

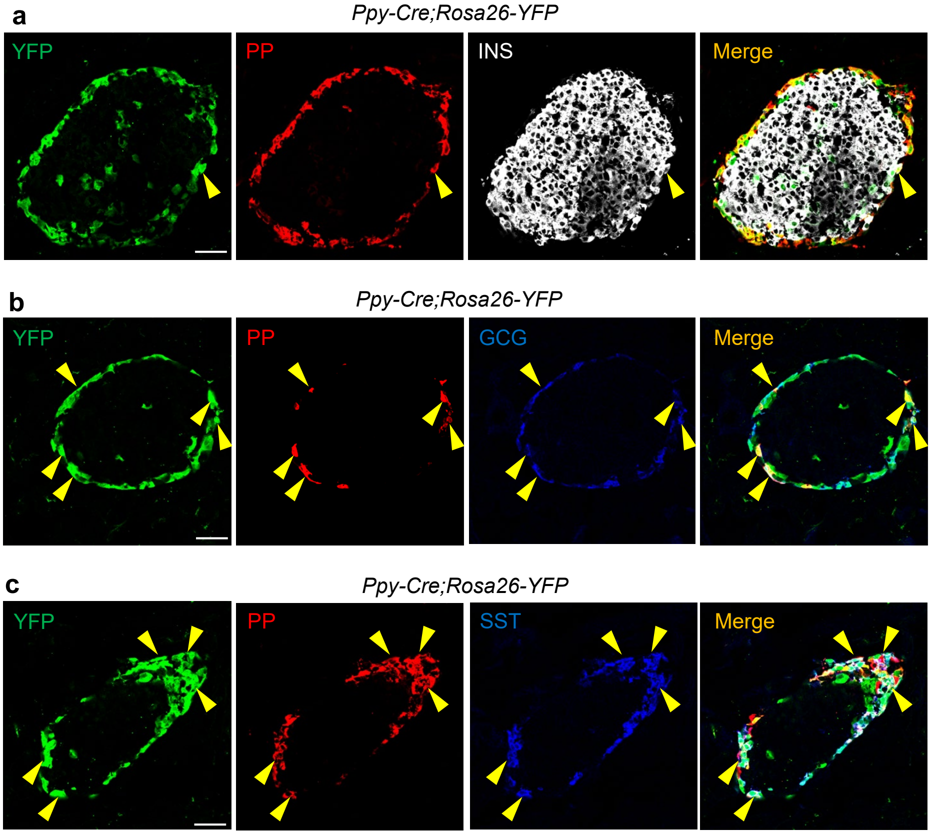

ESM Fig. 1 Some *Ppy*-lineage cells show bihormonal characteristics. (**a-c**) YFP^+ PP^+ INS^+ cells, YFP^+ PP^+ GCG^+ cells, and YFP^+ PP^+ SST^+ cells in the head of the pancreas of adult *Ppy-Cre;Rosa26-YFP* mice (yellow arrowheads for all). Scale bars, 50 µm.

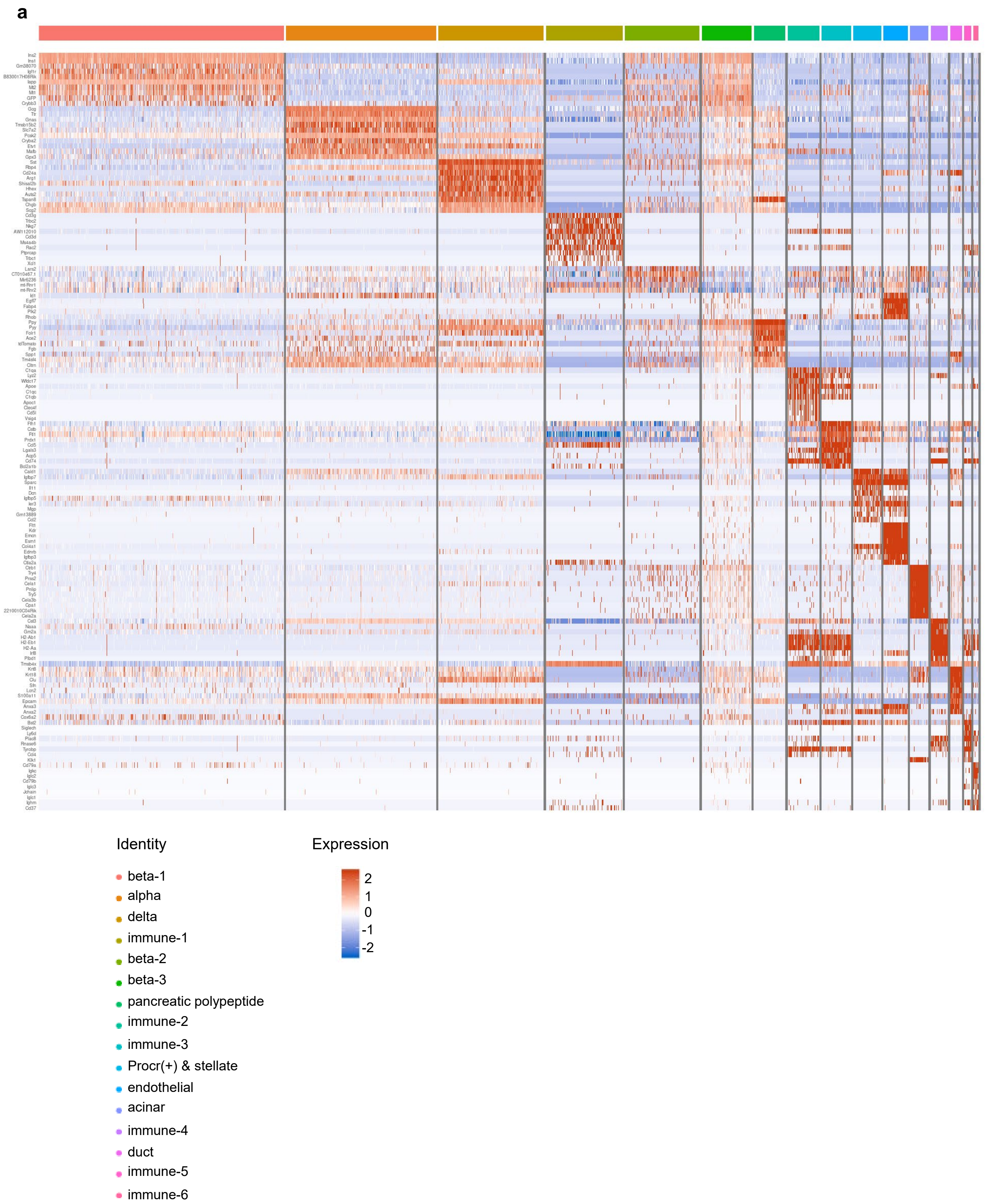
a
Identity
beta-1
alpha
delta
immune-1
beta-2
beta-3
pancreatic polypeptide
immune-2
immune-3
Procr(+) & stellate
endothelial
acinar
immune-4
duct
immune-5
immune-6
Expression
2
1
0
-1
-2

b

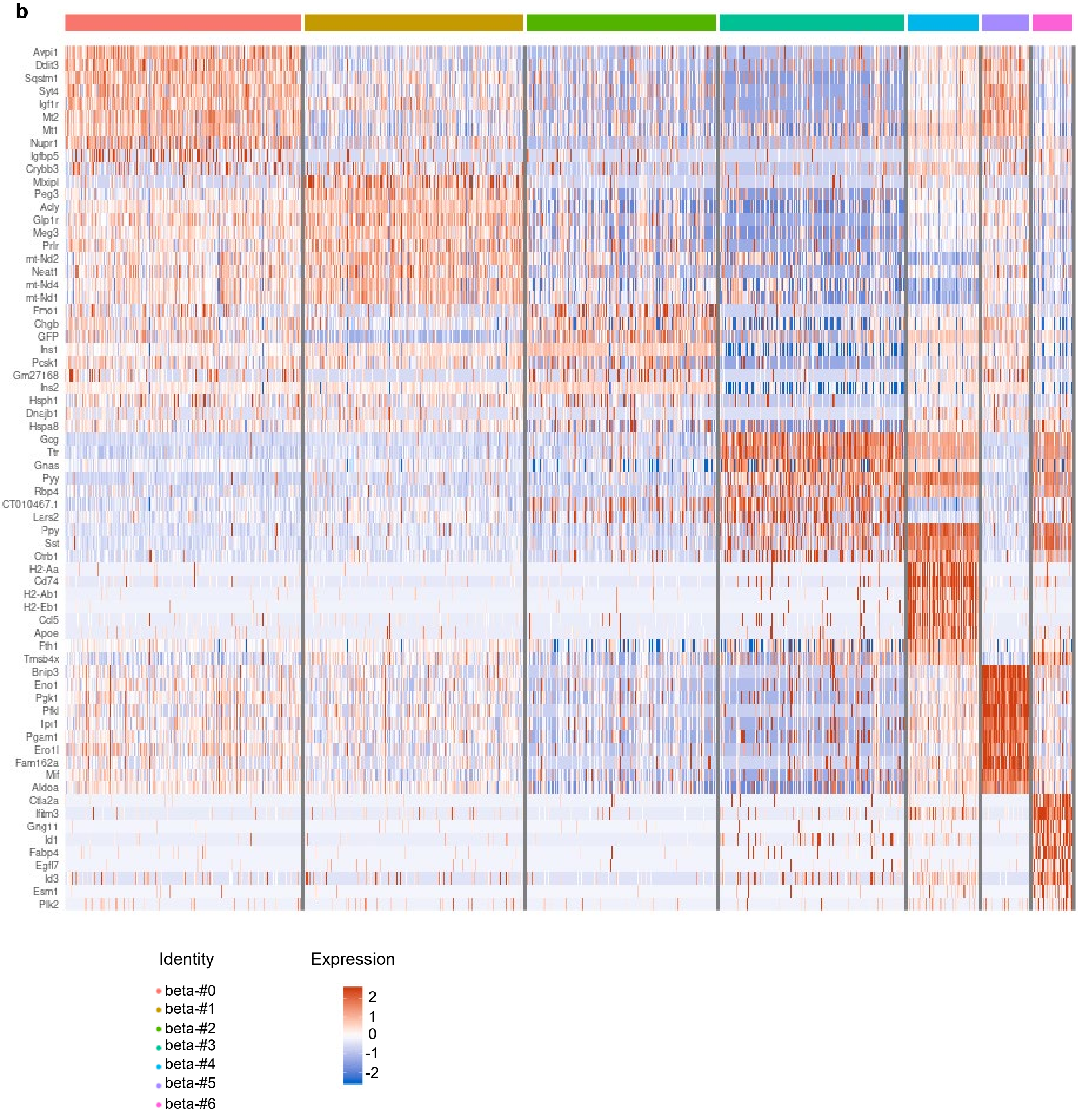

ESM Fig. 2 Heatmap analysis showing each cluster in islet cells and beta-cell subcluster with distinct signature genes. (**a**) Heatmap showing signature genes of each of the 16 islet cell clusters. Each column represents a single cell and each row represents one signature gene. The colour ranging from blue to red indicate low to high relative gene expression levels. (**b**) Heatmap showing signature genes of each of the 7 beta cell subclusters. Each column represents a single cell and each row represents one signature gene. The colour ranging from blue to red indicate low to high relative gene expression levels.

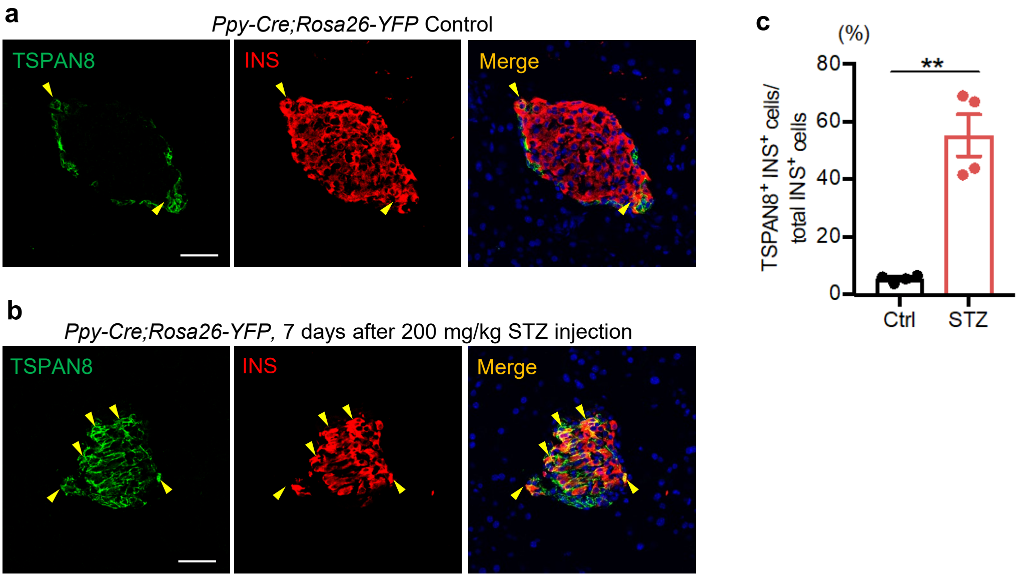

ESM Fig. 3 Characteristics of TSPAN-expressing beta cells in hyperglycaemic conditions. (**a**) Immunohistochemical analysis of TSPAN8$^+$ INS$^+$ cells in the head of the pancreas of *Ppy-Cre;Rosa26-YFP* mice 7 days after citrate buffer injection (arrowheads). Scale bar, 50 µm. (**b**) Immunohistochemical analysis of TSPAN8$^+$ INS$^+$ cells in the head of the pancreas of *Ppy-Cre;Rosa26-YFP* mice 7 days after 200 mg/kg STZ injection (arrowheads). Scale bar, 50 µm. (**c**) Ratio of TSPAN8$^+$ INS$^+$ cells to total INS$^+$ cells in the head of the pancreas of *Ppy-Cre;Rosa26-YFP* mice 7 days after 200 mg/kg STZ injection compared with control mice ($n = 4$). Data are shown as the mean ± SEM. $^{**}p<0.01$, (two-tailed Student's t test).

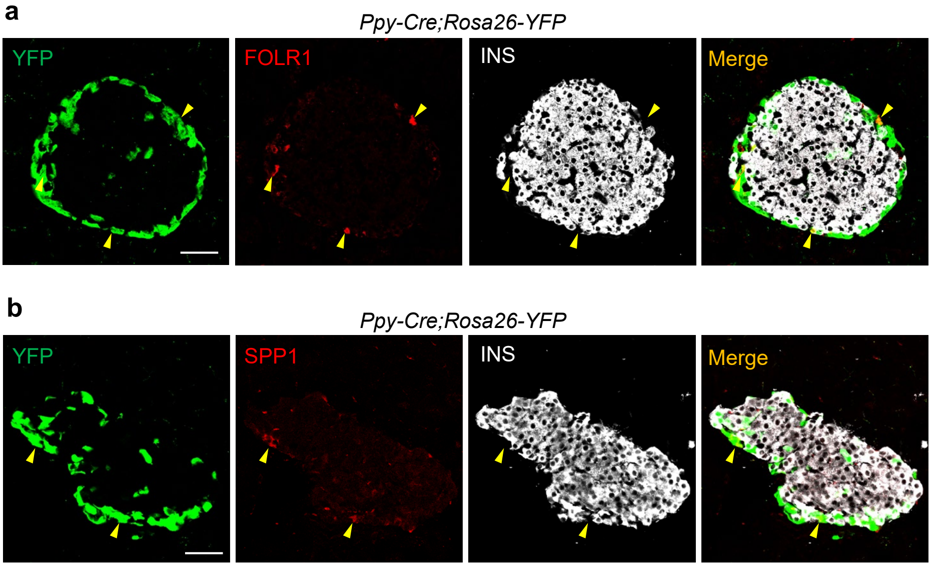

ESM Fig. 4 *Ppy*-lineage beta cells do not express FOLR1 and SPP1. **(a)** Immunohistochemistry of YFP$^+$ cells, FOLR1$^+$ cells, and INS$^+$ cells in the head of pancreas of *Ppy-Cre;Rosa26-YFP* mice. Arrowheads indicate YFP$^+$ FOLR1$^+$ INS$^-$ cells. Scale bar, 50 µm. **(b)** Immunohistochemistry of YFP$^+$ cells, SPP1$^+$ cells, and INS$^+$ cells in the head of pancreas of *Ppy-Cre;Rosa26-YFP* mice. Arrowheads indicate YFP$^+$ SPP1$^+$ INS$^-$ cells. Scale bar, 50 µm.
